# Combination of Genome-Wide Polymorphisms and Copy Number Variations of Pharmacogenes in Koreans

**DOI:** 10.3390/jpm11010033

**Published:** 2021-01-07

**Authors:** Nayoung Han, Jung Mi Oh, In-Wha Kim

**Affiliations:** College of Pharmacy and Research Institute of Pharmaceutical Sciences, Seoul National University, Seoul 08826, Korea; hans1217@gmail.com (N.H.); jmoh@snu.ac.kr (J.M.O.)

**Keywords:** polymorphisms, pharmacogenes

## Abstract

For predicting phenotypes and executing precision medicine, combination analysis of single nucleotide variants (SNVs) genotyping with copy number variations (CNVs) is required. The aim of this study was to discover SNVs or common copy CNVs and examine the combined frequencies of SNVs and CNVs in pharmacogenes using the Korean genome and epidemiology study (KoGES), a consortium project. The genotypes (*N* = 72,299) and CNV data (*N* = 1000) were provided by the Korean National Institute of Health, Korea Centers for Disease Control and Prevention. The allele frequencies of SNVs, CNVs, and combined SNVs with CNVs were calculated and haplotype analysis was performed. *CYP2D6* rs1065852 (c.100C>T, p.P34S) was the most common variant allele (48.23%). A total of 8454 haplotype blocks in 18 pharmacogenes were estimated. *DMD* ranked the highest in frequency for gene gain (64.52%), while *TPMT* ranked the highest in frequency for gene loss (51.80%). Copy number gain of *CYP4F2* was observed in 22 subjects; 13 of those subjects were carriers with CYP4F2*3 gain. In the case of *TPMT*, approximately one-half of the participants (*N* = 308) had loss of the TPMT*1*1 diplotype. The frequencies of SNVs and CNVs in pharmacogenes were determined using the Korean cohort-based genome-wide association study.

## 1. Introduction

It is well established that human genetic diversity is important for our understanding population histology [1], variability in disease susceptibility, and treatment response or adverse reactions to medications [2]. Single nucleotide variants (SNVs) are the most widely studied form of genetic variations and several SNVs have been linked to disease susceptibility and drug responses. Therefore, genome-wide association (GWA) studies have led to the identification of multiple genetic variants correlated with traits, such as body mass index, skin color [3], fat distribution [4], and glomerular filtration rate [5], and with diseases, such as autoimmune disease [6] and non-alcoholic fatty liver disease [7].

Additionally, these SNV markers from GWA studies can be used in pharmacogenomic research as a means of directly predicting interindividual responses to medicines [8]. Research has identified successfully the loci of genetic variants associated with responses to tumor necrosis factor inhibitors [9], antidepressants [10], and antipsychotics [11], and with adverse reactions induced by medicines, such as thiopurine-induced myelosuppression [12], statin-induced myopathy [13], and carbamazepine-induced hypersensitivity [14]. These genetic variations alter the structure and function of proteins such as drug-metabolizing enzymes, drug transporters, receptors, and response targets, collectively referred to as pharmacogenes [15].

Common copy number variations (CNVs) were estimated to occur in approximately 9.5% of the human reference genome and have non-random distribution [16]. CNVs account for at least five times more variable base pairs compared to that of SNVs when two human genomes are compared to each other [17,18]. As with SNVs, CNVs were found to influence susceptibility to cancer [19] as well as neurodegenerative disease [20] and psychiatric disease [21]. Despite their clinical significance, CNVs remain understudied compared to SNVs. The reasons may be that the detection of CNVs is more difficult and CNVs only occur with low-to-intermediate frequency [22]. However, for predicting phenotypes and executing precision medicine, combination analysis of SNVs genotyping with CNVs is required. There have been several studies to detect both CNVs and SNVs in *CYP2D6* [23,24]. However, CNV information integrated with polymorphisms on pharmacogenes is still not fully characterized [25]. Traditional methods are time-consuming and labor-intensive and a large number of participants are required.

The Korean genome and epidemiology study (KoGES) is a consortium project that was established as a genome epidemiological study for the research community with a health database and biobank to help investigate Korean population-based and gene–environment model studies [26,27,28]. Because this dataset contains a significant collection of SNVs and CNVs data from normal tissue and blood samples, KoGES is appropriate for combined pharmacogenomic studies. Thus, this study aimed to discover SNVs and CNVs and to examine the combined frequencies of SNVs and CNVs in pharmacogenes in the Korean population using this large public dataset.

## 2. Materials and Methods

### 2.1. Study Subjects

The study subjects were selected from the Ansan and Ansung study (*N* = 5836), the Health Examinee cohort (HEXA, *N* = 58,701), and the cardiovascular disease association study (CAVAS, *N* = 8105) that constitute the KoGES [29]. Epidemiologic data were provided by the Korean National Institute of Health, Korea Centers for Disease Control and Prevention (KCDC). Socio-demographic, medical history, health conditions, and family history of disease information were collected by trained interviewers using structured questionnaires. All physical examinations were administered by health professionals trained to follow standardized protocols. The participants who had cancer were excluded from the analysis. All subjects were middle-aged adults between 40 and 69 years of age. All study participants provided written informed consent.

### 2.2. Pharmacogenes

The pharmacogenomics-related genes were selected by the Very Important Pharmacogene summaries in the Pharmacogenomics Knowledge Base (as of March 2020) [30] and the Clinical Pharmacogenetics Implementation Consortium (CPIC) guideline (as of March 2020) [31]. The genes from the U.S. Food and Drug Administration (FDA) Table of Pharmacogenomic Biomarkers in Drug Labels (as of March 2020) were included [32]. A total of 191 genes were analyzed and are listed in Supplementary Table S1.

### 2.3. Data Collection and Preprocessing

The genotypes (*N* = 72,299) and CNV data (*N* = 1000) were provided by the KCDC. These imputated genotypes were produced by the Korea BioBank Array (referred to as KoreanChip, KCHIP, Seoul, The Republic of Korea) project, optimized for the Korean population [33]. A KCHIP array includes a total of 833,535 SNVs for autosomal chromosomes. Quality-controlled data were used for imputation analysis with 1000 Genomes Phase 3 data as a reference panel using ShapeIT v2 [34] and IMPUTE v2 [35]. An SNV missing rate greater than 0.05, SNVs with a minor allele frequency less than 0.01, or a Hardy–Weinberg equilibrium (HWE) of P less than 10^–6^ were excluded according to standard quality control procedures. The SNV position aligned to human reference genomes hg19 using the Bioconductor BiomaRt R package [36]. For each gene, 10 kb bases of region were added both upstream and downstream of the defined gene location. The CNV data of 1000 subjects were produced from the Ansan and Ansung study [37]. The CNV data were genotyped with the NimbleGen HD2 3 × 720 K comparative genomic hybridization array (aCGH) (Roche NimblGen, Madison, WI, USA) [37]. For the combination analysis of genotypes and CNVs, the variants from gene–drug pairs from CPIC were searched for their clinical effects. The functional effects of variants were predicted by SIFT (Sorting Intolerance From Tolerant) [38] and POLYPHEN-2 (Polymorphism Phenotyping v2) [39].

### 2.4. CNV Calling

R package that implements the Genome Alteration Detection Analysis algorithm (GADA) was used for CNV discovery [40]. To overcome the limitation of single algorithm detection, we tested different thresholds, T, from 3 to 8. CNV discovery with several parameters was tested to find the best parameters using known CNV regions [41]. Consequently, we selected the best parameter with high concordance with known CNV regions with T = 4.5, alpha = 0.2, and MinSegLen = 6. CNV regions longer than 50 bp in length were included for further analysis. A log 2 ratio cut-off of ±0.25 was used to define copy number gain and loss and cut-offs of ±0.8 were used to define amplification and deletion, respectively [42,43].

### 2.5. Data Analysis

Categorical variables such as gender and variant occurrences are presented in percentages and frequencies. Continuous variables such as age are presented with average and standard variations. The chi-squared test with one degree of freedom was used to test the departure from HWE for each variant. Data were analyzed with PLINK 1.9 or 2.0 [44] and R (version 3.6.3). Linkage disequilibrium analysis among pairs of SNVs was performed to identify the haplotype. Estimation of haplotype blocks and their frequencies were performed with PLINK and Haploview [45].

## 3. Results

### 3.1. Characteristics of the Study Population

For the KCHIP study, among the Ansan and Ansung study (*N* = 5493), HEXA (*N* = 58,701), and CAVAS (*N* = 8105), after excluding patients with cancer, 5182 of the Ansan and Ansung study subjects, 55,955 of HEXA, and 7890 of CAVAS remained. For the CNV data, 945 subjects remained after excluding patients with cancer. Among them, 614 subjects had SNV and CNV data. The characteristics of the subjects from the SNV and CNV data are presented in Table 1. The average ages of the subjects with SNV and CNV data were 54.08 and 54.05 years, respectively. The frequencies of female subjects (63.78%) was higher than that of male subjects (36.22%) in the SNV data, while that of female subjects (49.95%) was similar to that of male subjects (50.05%) in the CNV data.

### 3.2. Genotype Variants

**Figure 1:**
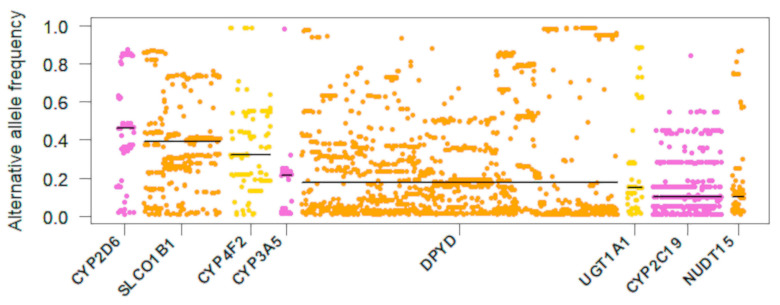
Single-nucleotide variants of pharmacogenes with alternative allele frequencies of more than 10% in a Korean population. Horizontal lines indicate median values.

A total of 36,853 SNVs in pharmacogenes were included for the further analysis. The allele frequencies of SNVs of more than 10% are listed in Supplementary Table S2. *VKORC1* rs9923231 (–1639G>A or G3673A) was found to be the most common alternative allele (92.42%). *CYP2D6* rs1065852 (c.100C>T, p.P34S) was the next common allele (48.23%). The allelic frequencies of CYP2C19*2 (rs4244285, c.681G>A, p.P227P) and CYP2C19*3 (rs4986893, c.636G>A, p.W212X) were 28.29% and 10.04%, respectively. The allelic frequency of CYP3A5*3 (rs776746, c.6986A>G) was 23.47%. CYP4F2*3 (rs2108622, c.1297C>T, p.V433M) and CYP4F2*2 rs3093105 (c.34T>G, p.W12G) were 32.41% and 13.40%, respectively. Among SNVs in pharmacogenes, those that were assigned as having level A evidence of gene-drug pairs by CPIC are shown in Figure 1. The median alternative allele frequency of *CYP2D6* variants was ranked the highest (46.17%, ranged from 1.02% to 87.34%), followed by *SLCO1B1* variants (39.32%, ranged from 1.07% to 86.62%). SNVs with frequencies less than 10% that were also assigned as having level A evidence of gene–drug pairs by CPIC or predicted to be deleterious by SIFT and POLYPHEN-2 are listed in Supplementary Table S3. *CACNA1S* rs3850625 (c.4615G>A (p.R1539C), *CFTR* rs121909046 (c.650A>G, p.E217G) and rs113857788 (c.4056G>C p.Q1352H), and *CYP2B6* rs8192709 (c.64C>T, p.R22C) were predicted to be deleterious by SIFT.

### 3.3. Haplotype Analysis

**Figure 2:**
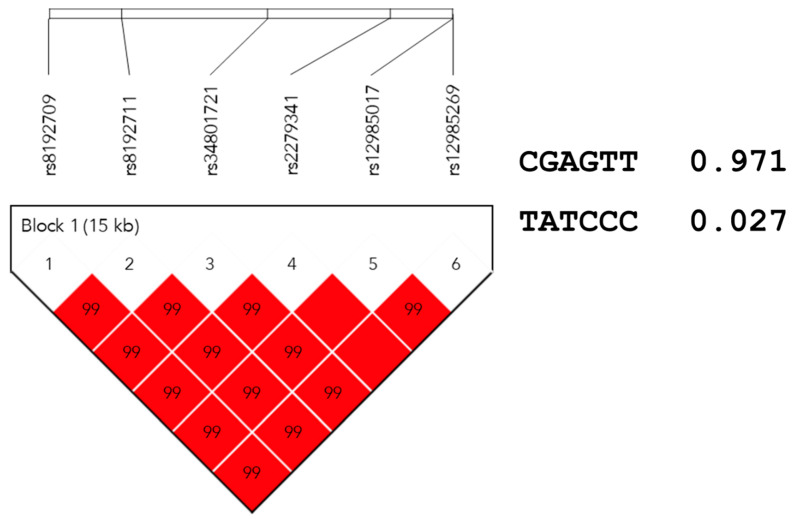
Haplotype block map constructed by candidate single-nucleotide variations in *CYP2B6*. Notes: Block 1 includes rs8192709, rs8192711, rs34801721, rs2279341, rs12985017, and rs12985269; the linkage disequilibrium between two SNPs is indicated by the standardized r^2^ (red boxes).

The frequency distributions of the variants or haplotypes were found to be significantly different among ethnic populations. Therefore, haplotype analysis was performed on about 18 pharmacogenomic genes from 73 gene–drug pairs with level A evidence by CPIC. A total of 8454 haplotype blocks in 18 genes were estimated, and the number varied from 2 to 3924 blocks per each gene, with an average of 4378. *CYP2B6* rs8192709 (c.64C>T, p.R22C) constructed a haplotype block with rs8192711 (G>A), rs34801721 (A>T), rs2279341 (G>C), rs12985017 (T>C), and rs12985269 (T>C) (Figure 2). The haplotype block of *CYP2B6* in Caucasians was constructed with rs2279341, rs12985017, and rs12985269. Carriers with the alternative haplotype T-A-T-C-C-C were found in 3.98% of this study population.

### 3.4. Copy Number Variation Profiling

**Figure 3:**
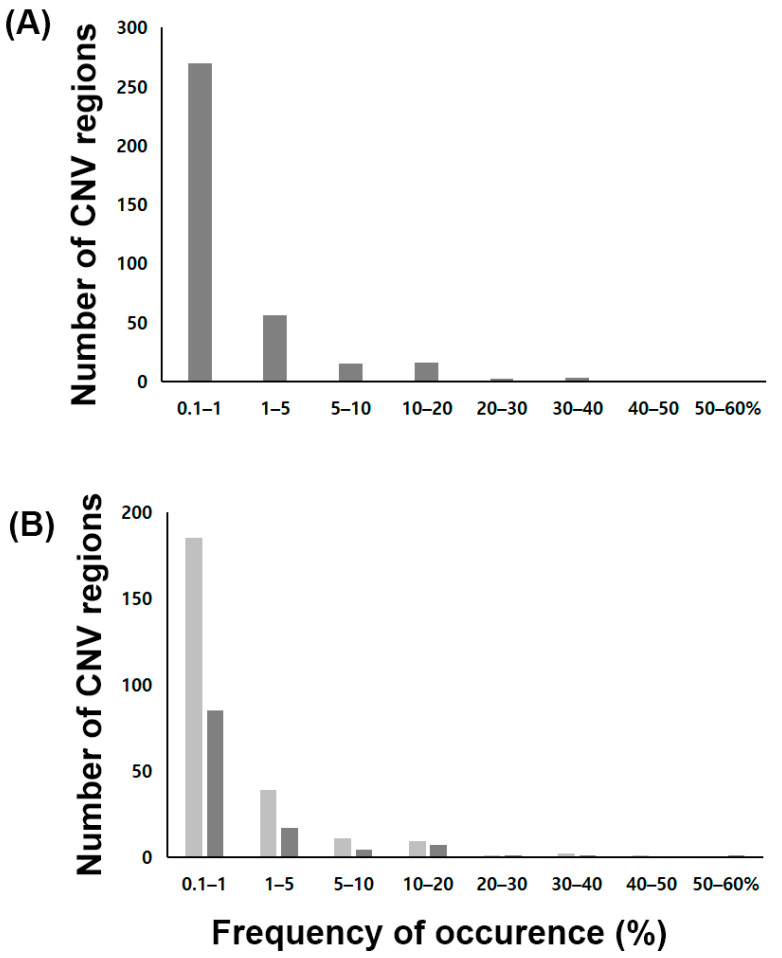
Distribution of copy number variation frequencies for the copy number variation regions in a Korean population. (**A**) Frequencies of copy number variation regions. (**B**) Copy number variation frequencies of the detected copy number variation regions, divided into gains and losses.

In the 947 subjects, segments with more than 1 CNV were determined in 937 subjects using GADA. In total, 448 segments were detected in 937 individuals with an average of 22.58 copy number segments in each individual. CNV regions of more than 50 bp were included for the further analysis. The mean and median lengths of these CNV regions were 4.29 and 2.21 kb, respectively. Figure 3 shows the distribution of the 333 CNV regions by frequency rate. Of the 333 CNV regions, 92 had frequency rates of >1%. The frequencies of CNVs were calculated and genes with a frequency of more than 1% are summarized in Table 2. *DMD* ranked the highest in frequency for gene gain (64.52%), while *TPMT* ranked the highest in frequency for gene loss (51.80%), and the frequency of *TPMT* deletion was 3.58%. There were gene gains in *G6PD* (17.21%), *KIT* (21.12%), and *OTC* (57.76%), while there was gene loss in *ABCB1* (15.31%), *BCR* (19.01%), *DMD* (20.27%), *EGFR* (41.39%), *HLA-B* (36.54%), *HLA-DRB1* (40.65%), *PDGFRA* (21.44%), and *SULT1A1* (19.75%) with a frequency of more than 10%. The genes with a CNV frequency of less than 1% are listed in Supplementary Table S4. Gene losses of *ABCG2* and *CYP2E1* were found in 0.63% of subjects, while the gene gain of *CYP2B6* was found in 0.21% of subjects.

### 3.5. Combination of Genotype Variants and CNVs

A total of 22 pharmacogenomic genes from 73 gene–drug pairs with level A evidence by CPIC were selected for the combination analysis of SNVs and CNVs in 614 subjects. CYP4F2*1*3 (24.43%) was most common *CYP4F2* diplotype followed by CYP4F2*2*3 (18.57%) (Table 3). Among the *CYP4F2* gains observed in 22 subjects, 13 subjects were carriers with a CYP4F2*3 gain. The frequency of *CYP4F2* loss was 0.49%. In the *TPMT* case, approximately half of the participants (*N* = 308) showed a loss of the TPMT*1*1 diplotype.

## 4. Discussion

Pharmacogenomic studies represent a critical component of precision medicine. Compared to SNVs, CNVs or the combined study of SNVs and CNVs all both relatively less studied. With regard to SNV or CNV data from genome epidemiological research, KoGES in Korea can be used for pharmacogenomic studies. The purpose of this study was to discover SNVs and CNVs and to examine the combined frequencies of SNVs and CNVs in pharmacogenes in Korea using KoGES.

For 191 pharmacogenes, a total of 36,853 SNVs from 69,027 subjects, 333 CNVs from 947 subjects, and combined data of SNVs and CNVs from 614 subjects were available in this study. The SNV rs9923231 (–1639G>A or G3673A) is known to alter a transcription factor binding site in the *VKORC1* promoter region, and this allele frequency in Asians was found to be approximately 0.92 [46], similar with our result. This variant was associated with decreased gene expression, resulting in decreased warfarin dose requirements. *CYP2D6* rs1065852 (c.100C>T, p.P34S) was the next most common allele (48.23%), and it appeared in *4, *10, *14A, and *36 alleles, with lower enzyme activities compared to the wild type [47]. This enzyme is involved in the metabolism of approximately 25% of commonly prescribed drugs, including antidepressants, antipsychotics, antiarrhythmics, β-blockers, and opioids [24]. The allelic frequencies of CYP2C19*2 and CYP2C19*3 were 28.29% and 10.04%, respectively, in our study, similar to earlier findings [48], and indicate that genomic data from the KoGES study are appropriate for pharmacogenomics studies in Korea. These losses of functional alleles of *CYP2C19* can increase the risks for serious cardiovascular events among patients treated with clopidogrel [49].

In our study, *CACNA1S* rs3850625 and *CFTR* rs121909046 and rs113857788 were predicted to be deleterious by SIFT. *CACNA1S* rs3850625 was associated with malignant hyperthermia accelerated by inhalational anesthetics and muscle relaxants [50]. Those two variants in the *CFTR* gene were found to have the strongest association with bronchiectasis and chronic pancreatitis in the Korean population [51].

According to a haplotype analysis, the haplotype block CYP2B6*2 (rs8192709) was constructed and the corresponding frequency was found to be 3.98 in this study. Approximately 3.4% of CYP2B6*2 variants were found in Han and Uygur Chinese [52]. Although the level of evidence for clinical annotations of CYP2B6*2 was lower than that for the CYP2B6*6 allele according to CPIC, this minor allele is known to decrease the clearance of methadone [53] or efavirenz [54].

The activities of several important drug-metabolizing genes, such as *CYP2B6*, *CYP2E1*, *CYP2D6*, *GSTM1*, and *SULT1A1*, are known to be related to variable copy numbers. In our study, CNVs of *CYP2B6*, *CYP2E1*, and *SULT1A1* were detected, whereas CNV data from KoGES did not cover *CYP2D6* and *GSTM1* genes. Accordingly, alternative methods during a CNV analysis are needed to detect those genes.

The *DMD* gene found to be the most frequent in terms of the copy number gain in our study is the largest gene in the human genome, encompassing 2.2 Mb and encoding for a muscular protein, dystrophin, which is related to the X-linked recessive disorders Duchenne muscular dystrophy and Becker muscular dystrophy [55]. Deletions or complex rearrangements usually occur between exons 43 and 55 or exons 2 and 23 [55]. Most carriers with mutations or deletions of the *DMD* gene are asymptomatic [56]. One hundred and seventeen different deletions and 48 duplications in the *DMD* gene were found in 507 Korean patients with Duchenne muscular dystrophy or Becker muscular dystrophy [57].

*TPMT* ranked highest here in terms of the frequency of gene loss at 3.58% in our results. This is most likely due to a variable number of tandem repeats (VNTR) within a G/C-rich region in the promotor of *TPMT* [58]. The frequency of the VNTR allele, consisting of two repeat sequence motifs A, one motif B, and one motif C, was reported to be 48.2% in an Asian British cohort [59]. The patterns and total number of VNTR alleles were associated with the level of TPMT activity [59]. The *TPMT* gene encoding thiopurine S-methyltransferase is a crucial enzyme during the metabolism of thiopurine drugs such as azathioprine and 6-mercoptopurine [60].

In the next step, the CNV data were combined with SNVs for pharmacogenes. The loss frequency of TPMT*3C (rs1142345, c.A719G, p.Y240C) was 1.79%. The TPMT*3C variant, with moderate activity, was found to be the most frequent alternative allele in Koreans, and TPMT deficiency can increase certain fatal adverse reaction risks, such as bone marrow toxicity and myelosuppression induced by 6-mercaptopurine [61]. Thiopurine-associated leukopenia (more than 30%) was found to be considerably higher than expected according to the frequency of the *TPMT* variant (~1%) in Koreans with Crohn’s disease [62]. This result may be related to the copy number variation in the *TPMT* gene. Despite the fact that less than 5% of the samples showed gene gains or losses in these genes, the corresponding clinical impacts should be considered when medicines associated with these genes are administered.

A limitation of this study was that the CNV frequencies of some genes differed from those in previous studies [63]. This difference may have been caused by the different assay methods. There are many different methods for determining the CNVs of genes, and each method has advantages and pitfalls. The array CGH methods and SNP arrays and CNV arrays are excellent for initial scans along the lines of the SNP GWA study, and other PCR-based methods such as multiplex ligation-dependent probe amplification (MLPA) are used for conformation to genotype copy numbers [64]. The KCHIP array did not contain SNVs for sex chromosomes, meaning that pharmacogenes such as *DMD* and *G6PD* could not be included in the analysis of the combinations of genotype variants and CNVs. Another limitation in our study was that hybrid pseudogene, conversion, or tandem alleles cannot be determined due to the assay method used in this study. Additionally, as subjects with common complex diseases such as diabetic mellitus, hypertension, and cardiovascular disease were not excluded, this could affect the results of this study. Further studies with regard to functional variation evaluations and associated determinations are needed to manage patients more efficiently.

The 1000 Genomes Project and the Encyclopedia of DNA Elements Project produced comprehensive maps outlining the regions of the human genome containing SNVs, multi-nucleotide variants, and CNVs [65]. However, combination analyses of SNVs with CNVs in pharmacogenetic studies are limited. Here, we conducted a combined analysis of SNVs with CNVs in pharmacogenes in Koreans.

In conclusion, the frequencies of SNVs and CNVs in pharmacogenes were determined by means of a Korean cohort-based GWA study. Though further assessments of correlations with phenotype changes are necessary, the results here may be useful for the identification of genetic causes of cases involving severe drug-induced toxicity or reduced therapeutic benefits from a drug.

## Figures and Tables

**Table 1:** Demographic characteristics of study subjects.

Characteristics	SNV	CNV	Combination of SNV with CNV
Number of patients, *n*	69,027	947	614
Age, years	54.08 ± 8.31	54.05 ± 9.08	52.82 ± 8.80
Gender			
male	25,004 (36.22)	474 (50.05)	311 (50.65)
female	44,023 (63.78)	473 (49.95)	303 (49.35)

Values are reported as *n* (%) or mean ± standard deviation; SNV, single nucleotide variation; CNV, copy number variation.

**Table 2:** Copy number variations for pharmacogenes with a frequency of more than 1% in Koreans.

Gene	Position	Gain Frequency (%)	Loss Frequency (%)
*ABCB1*	7: 87133179−87342639	0.11	15.31
*ALK*	2: 29415640−30144477	6.12	1.06
*ALOX5*	10: 45869624−45941567	6.65	1.58
*BCR*	11: 23522552−23660224	0.11	19.01
*BRCA*	17: 41196312−41277500	2.22	2.64
*COMT*	19: 19929263−19957498	7.07	0.32
*CYP2A6*	19: 41349443−41356352	1.27	1.48
*CYP4F2*	19: 15988834−16008884	3.80	0.42
*DMD*	X: 31137345−33229673	64.52	20.27
*EGFR*	7: 55086725−55275031	2.32	41.39
*ESR1*	6: 152128814−152424408	0	1.48
*G6PD*	X: 153759606−153775233	17.21	0.42
*HLA-B*	6: 31237743−31324989	0.42	36.54
*HLA-DRB1*	6: 32489683−32557613	0.32	40.65
*KIT*	4: 55524095−55606881	21.12	2.22
*OTC*	X: 38211736−38280703	57.76	0.42
*PDGFRA*	4: 55095264−55164412	0.11	21.44
*RYR1*	19: 38924340−39078204	5.07	2.11
*SMN2*	5: 70220768−70248842	2.11	0.11
*SULT1A1*	16: 28616908−28620649	7.71	19.75
*TPMT*	6: 18128545−18155374	0	51.80

**Table 3:** Copy number variation combined with single nucleotide variations in Koreans (*N* = 614).

Gene Allele	Subjects (*N*)	Frequency (%)
CYP4F2*1*1	258	42.02
CYP4F2*1*2	1	0.16
CYP4F2*1*3	150	24.43
CYP4F2*3*3	22	3.58
CYP4F2*2*3	114	18.57
CYP4F2*1*2-*3*3	31	5.05
CYP4F2*2*2-*3*3	13	2.12
CYP4F2*1*1 gain	9	1.47
CYP4F2*1*3 gain	6	0.98
CYP4F2*2*3 gain	6	0.98
CYP4F2*3*3 gain	1	0.16
CYP4F2*1*1 loss	2	0.33
CYP4F2*2*3 loss	1	0.16
TPMT*1*1	287	46.74
TPMT*1*3C	8	1.30
TPMT*1*1 loss	308	50.16
TPMT*1*3C loss	11	1.79

## Data Availability

The datasets generated during the current study are available from the corresponding authors on reasonable request.

## Supplementary Materials

The following are available online at https://www.mdpi.com/2075-4426/11/1/33/s1, Table S1: List of 129 pharmacogenes in this study. Table S2: Single nucleotide variants of pharmacogenes with an allele frequency of more than 10% in Koreans. Table S3: Single nucleotide variants of pharmacogenes with an allele frequency of more than 10% in Koreans. Table S4. Copy number variations for pharmacogenes with a frequency of less than 1% in Koreans.
