# Supplementary material for: Combination of Genome-Wide Polymorphisms and Copy Number Variations of Pharmacogenes in Koreans
Source: J Pers Med. 2021 Jan 7;11(1):33. doi: 10.3390/jpm11010033 (PMC7825650; doi:10.3390/jpm11010033)
Supplement: Supplementary file 1 [file jpm-11-00033-s001.pdf]

Supplementary Table S1 List of 129 pharmacogenes in this study

Classification	Gene
Drug metabolizing enzymes	*ADH1A, ADH1B, ADH1C, ALDH1A1, BCHE, COMT, CYP1A2, CYP2A6, CYP2B6, CYP2C19, CYP2C8, CYP2C9, CYP2D6, CYP2E1, CYP2J2, CYP3A4, CYP3A5, CYP4F2, DPYD, G6PD, GSTP1, GSTT1, NAT2, SULT1A1, TPMT, UGT1A1*
Drug transporters	*ABCB1, ABCG2, SLC19A1, SLC22A1, SLCO1B1*
Targets/Pathway	*ABL1, ACE, ADRB1, ADRB2, ALK, ALOX5, ASS1, BCR, BRAF, BRCA1, CACNA1S, CD274, CFTR, CPOX, CPS1, CYB5R1, DMD, DRD2, EGFR, ERBB2, F2, F5, FGFR1, FIP1L1, FLT3, GALNS, GLA, HBB, HMBS, HMGCR, HPRT1, IDH1, IDH2, IGH, IL12A, IL12B, IL23A, IL28B, IL2RA, KCNH2, KCNJ11, KIT, KRAS, MS4A1, MTHFR, NAGS, NECTIN4, NPM1, NQO1, NRAS, NTRK1, NUDT15, OTC, P2RY12, P2RY1, PDGFRA, PDGFRB, PGR, PIK3CA, PPOX, PRF1, PROC, PROS1, PTGIS, PTGS2, RAB27A, RET, ROS1, SCN5A, SERPINC1, SH2D1A, SMN2, STX11, STXBP2, TNFRSF8, TPP1, TTR, TYMS, UNC13D, VKORC1, XIAP*
gDNA repair	*POLG*
Transcription factor	*AHR, ESR1, NR1I2, MYCN, PML, RARA, RYR1, TP53, VDR*
Miscellaneous	*HLA-A, HLA-B, HLA-DQA1, HLA-DRB1*

Supplemental Table S2 Single nucleotide variants of pharmacogenes with an allele frequency of more than 10% in Koreans

Gene	rsID	position	Variant	Function	Minor allele Frequency (%)	HWE P value
CYP2C19	rs12769205	10:96535124	A>G	intron	28.29	0.4058
CYP2C19	rs4986893	10: 96540410	G>A	stop gain	10.04	0.1807
CYP2C19	rs4244285	10: 96541616	G>A	synonymous	28.29	0.4058
CYP2D6	rs1065852	22:42526694	G>A	missense	48.23	0.4994
CYP2D6	rs16947	22:42523943	G>A	missense	14.96	0.2544
CYP2D6	rs1135840	22:42522613	G>C	missense	36.43	0.4632
CYP3A5	rs776746	7: 99270539	C>T	splice acceptor	23.47	0.3593
CYP4F2	rs3093105	19: 16008388	T>G	missense	13.40	0.2320
CYP4F2	rs2108622	19: 15990431	C>T	missense	32.41	0.4381
DPYD	rs72728438	1: 97847874	T>C	intron	19.25	0.3109
NUDT15	rs116855232	13: 48619855	C>T	missense	10.52	0.1883
SLCO1B1	rs4149015	12: 21283322	G>A	upstream	14.28	0.2449
SLCO1B1	rs2306282	12: 21329802	G>A	missense	26.72	0.3916
SLCO1B1	rs4149056	12: 21331549	T>C	missense	14.26	0.2445
SLCO1B1	rs4149057	12: 21331599	T>C	synonymous	25.97	0.3845
SLCO1B1	rs2291075	12: 21331625	C>T	synonymous	43.45	0.4914
UGT1A1	rs4124874	2: 234665659	T>G	intron	28.20	0.4050
UGT1A1	rs887829	2: 234668570	C>T	intron	12.27	0.2152
UGT1A1	rs4148323	2: 234669144	G>A	missense	19.33	0.3119
VKORC1	rs9923231	16:31107689	T>C	upstream	92.42	0.1400

HWE, Hardy-Weinberg Equilibrium

Supplementary Table S3. Single nucleotide variants of pharmacogenes with an allele frequency of less than 10% in Koreans

Gene	rsID	position	Variant	SIFT Function	Alternative allele Frequency (%)	HWE P value
CACNA1S	rs3850625	1: 201016296	G>A	Deleterious	3.89	0.075
CFTR	rs121909046	7: 117175372	A>G	Deleterious	2.27	0.864
CFTR	rs113857788	7: 117304834	G>C	Deleterious	1.88	0.410
CYP2B6	rs8192709	19: 41497274	C>T	Deleterious	2.73	0.942
CYP2C19	rs12248560	10:96521657	C>T	–	1.12	0.297
CYP2C9	rs1057910	10:96741053	A>C	–	4.06	0.056
CYP2D6	rs28371725	22:42523805	C>T	–	2.29	0.267
TPMT	rs1142345	6:18130918	T>C	–	1.56	0.618
UGT1A1	rs35350960	2:234669619	C>A	–	1.22	0.874
VKORC1	rs7294	16:31102321	C>T	–	7.56	0.252

HWE, Hardy-Weinberg Equilibrium

Supplementary Table S4. Copy number variations for pharmacogenes with a frequency of less than 1% in Koreans

Gene	Position	Gain Frequency (%)	Loss Frequency (%)
DPYD	1: 97543300−98386615	0.11	0.11
F5	1: 169481192−169555769	0.84	0
PIK3CA	3: 178866311−178952497	0.11	0
ABCG2	4: 89011416−89080011	0	0.63
ADH1B	4: 100227527−100242572	0.21	0
PDGFRB	5: 149493402−149535422	0.11	0.11
AHR	7: 17338276−17385775	0.21	0
BRAF	7: 140433813−140624564	0	0.11
CD274	9: 5450503−5470567	0.11	0
ABL1	9: 133589268−133763062	0.63	0
IL2RA	10: 6052657−6104333	0.11	0.21
CYP2E1	10: 135340867−135352620	0	0.63
MS4A1	11: 60223282−60238225	0.11	0
PGR	11: 100900355−101000544	0.11	0
NAGS	17: 42082032−42086436	0	0.11
CYP2B6	19: 41497204−41524301	0.21	0
PTGIS	20: 48120411−48184707	0.95	0
